# Shuni Virus as Cause of Neurologic Disease in Horses

**DOI:** 10.3201/eid1802.111403

**Published:** 2012-02

**Authors:** Charmaine van Eeden, June H. Williams, Truuske G.H. Gerdes, Erna van Wilpe, Adrianne Viljoen, Robert Swanepoel, Marietjie Venter

**Affiliations:** University of Pretoria, Pretoria, South Africa (C. van Eeden, J.H. Williams, E. van Wilpe, A. Viljoen, R. Swanepoel);; Onderstepoort Veterinary Institute, Pretoria (T.G.H. Gerdes);; National Institute for Communicable Diseases, Sandringham, South Africa (M. Venter)

**Keywords:** Orthobunyavirus, Shuni virus, neurological, horses, viruses

## Abstract

To determine which agents cause neurologic disease in horses, we conducted reverse transcription PCR on isolates from of a horse with encephalitis and 111 other horses with acute disease. Shuni virus was found in 7 horses, 5 of which had neurologic signs. Testing for lesser known viruses should be considered for horses with unexplained illness.

Several mosquito-borne alphaviruses, flaviviruses, and orthobunyaviruses, including West Nile, Rift Valley fever, and chikungunya viruses, with zoonotic potential have emerged from Africa to cause major outbreaks in previously unaffected areas (*1*). Horses are highly sensitive to some of these viruses and have been used as sentinels for the identification of arboviruses associated with neurologic disease in South Africa (*2*). During the seasonal occurrence of common vector-borne diseases such as African horse sickness and equine encephalosis, many horses have febrile, neurologic, and fatal infections for which the etiology remains undetermined.

We report a case in which a virus isolated in cell culture from the brain of a euthanized horse that had severe encephalitis was identified as Shuni virus (SHUV), a member of the family Bunyaviridae, genus Orthobunyavirus, serogroup Simbu. SHUV-specific primers were designed and used to perform reverse transcription PCRs (RT-PCRs) on specimens from an additional 111 horses with fever and nervous disease that had been screened for the more common pathogens over 18 months. The study was conducted in accordance with the recommendations of the Faculty of Health Sciences Ethics Committee of the University of Pretoria under protocols 129/2006 and H016-09.

## The Study

**Figure 1:**
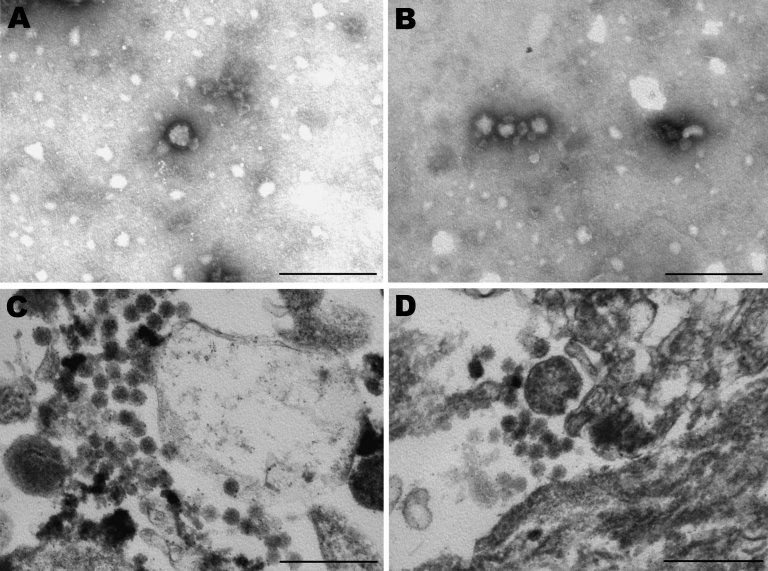
Electron micrographs of Vero cells infected with virus from horse SAE 18/09. A, B) Negative stain showing fringed particles (bunyavirus size) with bleb formation. C, D) Resin section showing spherical and pleomorphic bunyavirus particles in the range of 80–100 nm. Scale bars = 250 nm.

In January 2009, a crossbreed yearling horse (case SAE 18/09) was found wandering aimlessly in its paddock in the Vaalwater District of Limpopo Province, South Africa. The horse became progressively ataxic and, when recumbent, was referred to the hospital at the Faculty of Veterinary Science, University of Pretoria. When examined, the horse was unaware of its surroundings and paddled constantly (front legs swinging inward in their trajectory). Sedation, including the use of ketamine as a last resort, failed to calm the animal. The yearling experienced several episodes of muscle spasm interspersed with tremors and was euthanized when its condition was deemed terminal. Cytologic examination of a cerebrospinal fluid sample taken at euthanasia found pleocytosis with 98% lymphocytes, suggestive of viral infection. An autopsy was performed, and various specimens were submitted for histopathologic and virologic examination. A cytopathic agent isolated from the brain of the horse could not be identified as one of the common horse pathogens, but electron microscopic examination of negative-stained preparations of culture fluid and resin sections of infected cells showed 80-nm to100-nm particles resembling bunyaviruses (Figure 1).

RNA was extracted from infected cultures by using the QIAamp Viral RNA Mini Kit (QIAGEN, Valencia, CA, USA). RT-PCRs were performed by using the Titan One Tube RT-PCR Kit (Roche, Mannheim, Germany) with published primers Bunya 1 and 2, which amplify a 550-bp fragment of the N gene of the S RNA segment of orthobunyaviruses (*3*). Blast search analysis (http://blast.ncbi.nlm.nih.gov/Blast.cgi) of the fragment showed that the amplicon was related to members of the Simbu serogroup of the genus Orthobunyavirus, and neighbor-joining phylogenetic analysis indicated high bootstrap support (>91%) for placement of SAE 18/09 within the Shuni, Aino, and Kaikular branches of the serogroup. The isolate shared 95.9% identity with SHUV and 91.1% identity with Aino virus (results not shown).

SHUV-specific amplification was then performed by using SHUV-specific primers designed for the present study, i.e., SHUVS111+ (5′-CGA TAC CGT TAG AGT CTT CTT CC-3′) and SHUVS688- (5′-CGA ATT GGG CAA GGA AAG T-3′). Nested PCRs were performed by using primers SHUVS178+ (5′-CCG AGT GTT GAT CTT ACA TTT GGT-3′) and SHUVS611- (5′-GCT GCA CGG ACA GCA TCT A-3′) and the Expand High FidelityPLUS PCR System (Roche, Mannheim, Germany) to produce 430-bp amplicons. Sequences were edited by using Sequencher version 4.6 (Gene Codes Corp., Ann Arbor, MI, USA) and aligned by using the ClustalW subroutine (www.ebi.ac.uk/Tools/msa/clustalw2/), which forms part of the Bioedit program (www.mbio.ncsu.edu/BioEdit/BioEdit.html). Phylogenetic trees were generated by using maximum-likelihood estimation in PhyML (http://code.google.com/p/phyml/) with 100 bootstrap replicates. P-distance analyses were carried out for nucleotide and amino acid sequences by using MEGA4 www.megasoftware.net).

Specimens from an additional 111 horses were submitted by veterinarians throughout South Africa, the Onderstepoort Veterinary Institute, and the Faculty of Veterinary Science, University of Pretoria, to the Department of Medical Virology, University of Pretoria, for investigation of febrile or nervous disease. These specimens were included in the SHUV study. Specimens had been screened as appropriate for poisons and specific pathogens, including rabies, equine herpes virus, African horse sickness virus, and equine encephalosis virus (*4**,**5*). Specimens found to be negative were subjected to RT-PCRs with alphavirus and flavivirus generic primers and West Nile virus–specific and SHUV-specific primers (*6**,**7*).

SHUV infection was identified in 7 horses, 2 (8%) of 26 with unexplained fever and 5 (6%) of 86 with nervous disease (Table). In 1 of the SHUV-infected horses with febrile illness, horse SAE 38/10 (Table), co-infection with an alphavirus was found, and the virus was identified as Middelburg virus. Three of the 5 horses that showed signs of nervous disease had to be euthanized when they were near death. The 2 horses with febrile disease and 2 with mild nervous disease recovered fully.

**Table:** Clinical signs in horses with Shuni virus infection, South Africa, 2009–2010

Case no.	Age, y	District, province	Specimen	Date received	Clinical signs	Outcome
SAE 18/09	1	Vaalwater, Limpopo	Brain, cell culture	2009 Jan 16	Ataxia, tremors, convulsions, recumbent with paddling of legs	Euthanized
SAE 72/09	5	Bapsfontein, Gauteng	Brain	2009 Jul 2	Depression, anorexia, ataxia, recumbent with paddling of legs	Euthanized
SAE 27/10	18	Unrecorded, Gauteng	Blood	2010 Apr 13	Tremors, petechiae, quadriplegia	Euthanized
SAE 38/10*	4	Kimberly, Northern Cape	Blood	2010 Apr 14	Fever, anorexia, leukopenia	Survived
SAE 39/10	4	Kimberly, Northern Cape	Blood	2010 Apr 14	Fever, anorexia, leukopenia	Survived
SAE 48/10	4	Norvalspont, Northern Cape	Blood	2010 Apr 23	Depression, anemia, icterus, hepatitis, anorexia, ataxia, partial paresis	Survived
SAE 109/10	13	Bronkhorstspruit, Gauteng	Blood	2010 Jul 27	Depression, anorexia, ataxia, tremors, hyperaesthesia	Survived

*Co-infected with Middelburg virus.

**Figure 2:**
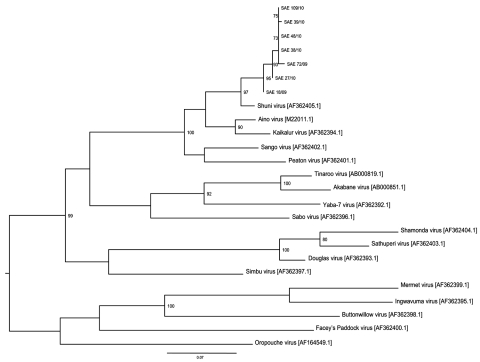
Maximum-likelihood tree constructed under the HKY codon position substitution model using PhyML (http://code.google.com/p/phyml/) of a 330-bp fragment of small segment RNA of Shuni virus (SHUV) identified in horses in South Africa, with representative sequences of selected other orthobunyaviruses. Scale bar = 0.07 nt substitutions. Estimates were based on bootstrap resampling conducted with 100 replicates. Only values >70 are shown. All SHUV amplicons were sequenced, and the data were deposited in GenBank, accession nos. SAE 18/09–HQ610137, SAE 72/09–HQ610138, SAE 27/10–HQ 610139, SAE 38/10–HQ 610140, SAE 39/10–HQ 610141, SAE 48/10–HQ 610142, and SAE 109/10–HQ 610143. Reference strains and GenBank accession numbers are indicated.

Maximum-likelihood analysis of 330-nt fragments of the amplicons with corresponding sequences of representatives of the Simbu serogroup of orthobunyaviruses confirmed that all strains clustered with SHUV virus (Figure 2). The nucleotide sequences differed from the prototype SHUV isolate by 3.6%–4.8% (average 4.2%) and from each other by 0%–1.8% (average 0.7%). The full N gene was determined for the isolate from horse SAE 18/09, and analysis showed it to differ from the original Shuni isolate by 2.9% and to Aino virus by 6.2% at the nucleotide level.

## Conclusions

SHUV was first isolated in the 1960s from cattle and sheep in abattoirs (*Cuilicoides* spp. midges tested as part of arbovirus surveys and in 1 instance from a febrile child in Nigeria) (*8**–**10*). Subsequently, the virus was isolated from pools of *Culex theileri* mosquitoes caught near Johannesburg and from cattle and a goat in KwaZulu-Natal Province, South Africa (*11**,**12*). In 1977, the virus was isolated from the brains of 2 horses that died of nervous disease, 1 in South Africa and 1 in Zimbabwe (*13**,**14*). Despite these data, no further investigations were undertaken to determine the role of the virus as a cause of neurologic disease in humans or animals. Identification of SHUV from a horse with severe neurologic signs prompted us to design specific Shuni virus primers and screen further cases of acute disease.

Over 18 months we identified 7 cases of SHUV infection, 5 of which were associated with neurologic signs. Our findings suggest that the role of SHUV as a pathogen may be underestimated and that it should be investigated routinely as a possible cause of unexplained nervous disease in humans and other animals in Africa. Most cases were identified in the autumn and winter months, which overlap with African horse sickeness, equine encephalosis, and West Nile virus outbreaks in South Africa (*5**,**15*), which have similar clinical signs. Such overlaps may contribute to the underrecognition of lesser known viruses, such as SHUV, because routine diagnostic investigation is limited to the more common viruses.

The discovery of a co-infection with Middelburg virus in 1 of the horses implies that broad screening for arbovirus infections in unexplained illnesses is warranted, and consideration should be given to inclusion of generic RT-PCRs for alphaviruses, flaviviruses, othobunyaviruses, and vesiculoviruses, in addition to African horse sickness and equine encephalosis viruses in future studies. Moreover, the inclusion of tests for immune response would improve the success rate for establishing diagnoses because viremia is fleeting in most arbovirus infections.
